# Haploidentical allograft is superior to matched sibling donor allograft in eradicating pre-transplantation minimal residual disease of AML patients as determined by multiparameter flow cytometry: a retrospective and prospective analysis

**DOI:** 10.1186/s13045-017-0502-3

**Published:** 2017-07-04

**Authors:** Ying-Jun Chang, Yu Wang, Yan-Rong Liu, Lan-Ping Xu, Xiao-Hui Zhang, Huan Chen, Yu-Hong Chen, Feng-Rong Wang, Wei Han, Yu-Qian Sun, Chen-Hua Yan, Fei-Fei Tang, Xiao-Dong Mo, Kai-Yan Liu, Xiao-Jun Huang

**Affiliations:** 1 Beijing Key Laboratory of Hematopoietic Stem Cell Transplantation, Peking University People’s Hospital & Peking University Institute of Hematology, No. 11 South Street of Xizhimen, Xicheng District, Beijing, 100044 People’s Republic of China; 2 Peking-Tsinghua Center for Life Sciences, Beijing, 100871 China

**Keywords:** Acute myeloid leukemia, Allogeneic stem cell transplantation, Minimal residual disease, Multiparameter flow cytometry, Unmanipulated haploidentical allografts

## Abstract

**Background:**

This study compared the effects of pre-transplantation minimal residual disease (pre-MRD) on outcomes in AML patients who underwent human leukocyte antigen-matched sibling donor transplantation (MSDT) or who received unmanipulated haploidentical allografts.

**Methods:**

A retrospective study (*n* = 339) and a prospective study (*n* = 340) were performed. MRD was determined using multiparameter flow cytometry.

**Results:**

Either after retrospective or prospective analysis, patients with negative pre-MRD (pre-MRDneg) had a lower incidence of relapse than those with positive pre-MRD (pre-MRDpos) in MSDT settings (*P* < 0.001 for all), but relapse was comparable in Haplo-SCT settings for patients with pre-MRDneg versus pre-MRDpos (*P* = 0.866 and 0.161, respectively). In either the retrospective (*n* = 65) or the prospective study (*n* = 76), pre-MRDpos subjects receiving Haplo-SCT experienced a lower incidence of relapse than those who underwent MSDT (*P* < 0.001 and *p* = 0.017, respectively). Of the patients with pre-MRDpos in either the total (*n* = 141) or the subgroup excluding cases which received donor lymphocyte infusion (DLI; *n* = 105), those who underwent MSDT had a higher incidence of relapse than those receiving haplo-SCT (*P* < 0.01 for all). Multivariate analysis showed that, for pre-MRDpos cases, haplo-SCT was associated with a low incidence of relapse and with better LFS and OS in either retrospective group, prospective group, combination groups, or subgroup not including cases which received DLI.

**Conclusions:**

The results indicated that, for pre-MRD-positive AML patients, haplo-SCT was associated with lower incidence of relapse and better survival, suggesting a stronger anti-leukemia effect.

**Electronic supplementary material:**

The online version of this article (doi:10.1186/s13045-017-0502-3) contains supplementary material, which is available to authorized users.

## Background

Allogeneic stem cell transplantation (SCT) remains a powerful therapeutic modality for patients with acute myeloid leukemia (AML) [1–8]. The superior clinical outcomes of allogeneic SCT versus chemotherapy alone as post-remission treatment could be related to the graft-versus-leukemia (GVL) effects of recovered donor T cells. Over the last 10 years, T-cell-replete haploidentical SCT (haplo-SCT), especially unmanipulated haplo-SCT with anti-thymocyte globulin (ATG) [3, 9, 10] or with post-cyclophosphamide (PT/Cy) [3], is widely accepted as a viable alternative for patients without HLA-identical donors, and its outcomes may be comparable to those of HLA-identical sibling donor transplantation (MSDT) or unrelated donor transplantation (MUDT) [4, 9]. However, it remains unclear whether haplo-SCT have different anti-leukemia effects than other allografts [11].

Increasing evidence suggests that the presence of minimal residual disease (MRD) before and after transplantation, which is detectable by multiparameter flow cytometry (MFC), identifies a subgroup of patients that is at high risk of relapse [12–18]. Zhou et al. [15] reported that peri-SCT MRD dynamics, as determined by MFC, are associated with a high risk of leukemia relapse and poor outcomes. Nevertheless, studies have focused mainly on the association of flow-cytometry-detected MRD with the outcomes of AML patients who underwent HLA-matched sibling donor transplantation (MSDT), cord blood transplantation (CBT), and MUDT [14, 15, 19, 20].

Currently, there is little information about the effects of MRD on transplant outcomes in haplo-SCT settings. Our earlier work indicated that patients with refractory/relapsed leukemia who received haplo-SCT experienced a significantly lower cumulative incidence of relapse compared to those who underwent MSDT (26% vs. 49%, *P* = 0.008) [21]. This suggested a stronger GVL effect for haplo-SCT than for MSDT. There may be differences in the anti-leukemia effects of haplo-SCT vs. MSDT [21], so this study investigated both the asso ciation of MRD status with outcomes in haplo-SCT and MDST settings and also possible differences in the transplant outcomes of patients with positive pre-MRD (as determined by MFC) who underwent haplo-SCT versus MDST. Our results provide new evidence that unmanipulated haplo-SCT is superior to matched sibling donor transplantation in eradicating pre-transplantation MRD, indicating that unmanipulated haplo-SCT have stronger GVL effects.

## Methods

### Study design

**Fig. 1:**
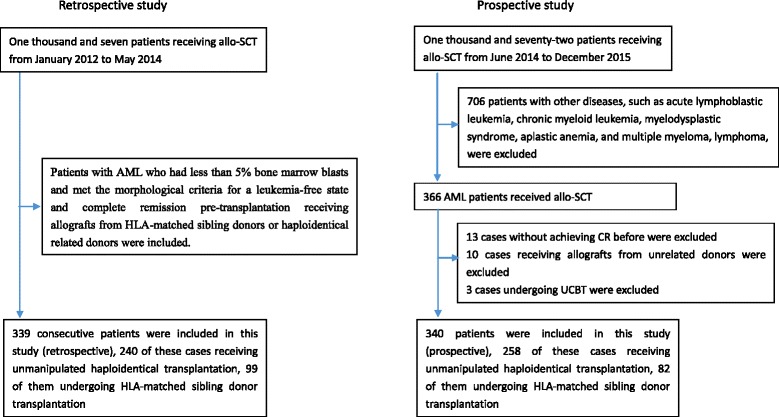
CONSORT (the Consolidated Standards of Reporting Trials) diagram

The retrospective analysis includes AML patients who were enrolled at the Peking University People’s Hospital between January 2012 and May 2014. The prospective study included AML patients who were recruited at the Peking University People’s Hospital between June 2014 and December 2015. All cases were treated according to our protocol, which is registered at http://www.chictr.org.cn/ as #ChiCTR-OCH-10000940 [4] (Fig. 1).

### Transplant protocol

Unmanipulated haplo-SCT and MSDT was performed according to the protocols reported previously by our group [4].

### Donor lymphocyte infusion

The indications for donor lymphocyte infusion (DLI) included hematological leukemia relapse, receiving chemotherapy followed by DLI, molecular test results that provided evidence of persistent leukemia or recurrence in subjects without graft-versus-host disease (GVHD), and graft failure (GF). The DLI protocol included two elements: (1) granulocyte colony-stimulating factor mobilized peripheral blood stem cells instead of steady-donor lymphocyte harvests were used and (2) a short-term immunosuppressive agent was used for prevention of DLI-associated GVHD. The median dose of mononuclear cells (MNC) for each infusion was 1.0 × 10^8^/kg. Subjects could receive up to four courses of DLIs. Subjects receiving DLIs from a haploidentical donor received cyclosporine (CSA) for 6 weeks after each infusion to prevent GVHD. Subjects receiving DLIs from a HLA-identical related donor received CSA or methotrexate (MTX) for 2–4 weeks after each infusion to prevent GVHD. In subjects receiving DLI from a HLA-identical related donor with prior ≥grade II acute GVHD or ≥moderate chronic GVHD received CSA after DLI whereas others received MTX. The starting dose of CSA was 2.5 mg/kg/day, and the dose was adjusted to maintain a plasma concentration 150–250 ng/ml. MTX, 10 mg, was given on days +1, +4, +8, +15, and +21 [22–24]. For relapse treatment, induction chemotherapy followed by DLI and GVHD prophylaxis was given. For relapse prophylaxis or GF, only DLI and GVHD prevention were used.

### MFC detection of MRD

Eight-color MFC was performed in all patients as a routine clinical test on bone marrow aspirate samples that were obtained as part of baseline assessment before SCT as well as around 30 to 180 days after transplantation according to previous studies [16, 18, 25, 26]. A panel of eight antibody combinations that recognize CD7, CD11b, CD13, CD14, CD16, CD19, CD33, CD34, CD38, CD41, CD45, CD56, CD61, CD64, CD71, CD117, CD123, and HLA-DR was used for MRD detection, and 0.2–1 million events per tube were acquired on a FACS Cant II. The isotype control monoclonal antibodies were used. Positive MRD was considered when a cluster of more than 25 cells with leukemia-associated immunophenotypes (LAIP) and SSC characteristics identified in all plots of interest and carrying at least two LAIP markers identified at diagnosis was observed. For those without LAIP markers at diagnosis, MRD was identified as a cell population showing deviation from the normal patterns of antigen expression seen on specific cell lineages at specific stages of maturation compared with either normal or regenerating marrow [27]. A lower limit of detection (LOD) of 0.01% was targeted. When abnormal cells were identified, the cells were quantified as a percentage of the total CD45^+^ white cell events. Any measurable level of MRD was considered positive. The standardized assays and quality controls were performed according to previous reports [28, 29]. The results of the MFC assessments of MRD were made available to the transplant teams. The significant level of MRD was set up by choosing a logarithmic scale that correlates with survival estimates and CIR as described previously [16, 30].

### Outcome

The primary study end point was the cumulative incidence of leukemia relapse. The secondary end points were the cumulative incidences of non-relapse mortality (NRM) and the probabilities of leukemia-free survival (LFS) and overall survival (OS).

Engraftment, GF, infection, NRM, relapse, LFS, and OS were defined as described previously [31]. Acute GVHD was defined and graded based on the pattern and severity of organ involvement [23]. Chronic GVHD was defined and graded according to the National Institute of Health criteria [32]. Relapse was defined based on histological criteria [23].

### Statistical analysis

Patient characteristics were compared between the MRDpos and MRDneg groups with the *χ* ^2^ statistic for categorical variables and the Mann–Whitney test for continuous variables. Cumulative incidence curves were used in a competing risk setting, with relapse treated as a competing event, to calculate NRM probabilities, and with death from any cause as a competing risk for GVHD, engraftment, and relapse. The time to GVHD was defined as the time from transplantation to the onset of GVHD of any grade. The probabilities of LFS and OS were estimated with the Kaplan–Meier method. MRD status pre- or post-transplantation and all variables in Table 1 were included in the univariate analysis. Only variables with *P* < 0.1 were included in a Cox proportional hazards model with time-dependent variables. Unless otherwise specified, *P* values were based on two-sided hypothesis tests. Alpha was set at 0.05. Most analyses were performed with SPSS 16.0 (Mathsoft, Seattle, WA, USA).

**Table 1:** Patient and donor characteristics in the retrospective study

Characteristics	All patients	MSDT			HBMT		
		MRDneg	MRDpos		MRDneg	MRDpos	
Number of patients	339	85	14		189	51	
Median age (range), years	31 (2–60)	41 (12–57)	44 (5–57)	0.457	27 (2–60)	28 (9–57)	0.151
Weight (range), kg	61 (15.5–118)	66.5 (29–97)	68 (23–96)	1.000	64 (15.5–118)	60 (25–102)	0.286
Male, *n* (%)	165 (48.7%)	41 (48.2%)	9 (64.3%)	0.263	96 (50.8%)	19 (37.3%)	0.086
Diagnosis, *n* (%)				0.264			0.031
De novo AML	333 (98.2%)	84 (98.8%)	13 (92.9%)		188 (99.5%)	48 (94.1%)	
Secondary AML	6 (1.8%)	1 (1.2%)	1 (7.1%)		1 (0.5%)	3 (5.9%)	
Disease status, *n* (%)				0.217			0.137
CR1	301 (88.8%)	81 (95.3%)	12 (85.7%)		167 (88.4%)	41 (80.4%)	
CR > 1	38 (11.2%)	4 (4.7%)	1 (14.3%)		22 (11.6%)	10 (19.6%)	
FLT3-ITD mutation				0.302			0.582
Yes	20 (5.9%)	5 (5.9%)	2 (14.3%)		11 (5.8%)	2 (3.9%)	
No	319 (94.1%)	80 (94.1%)	12 (85.7%)		178 (94.2%)	49 (96.1%)	
Cytogenetics				0.247			0.285
Favorable	59 (17.4%)	16 (18.8%)	1 (7.1%)		30 (15.9%)	12 (23.5%)	
Intermediate	263 (77.6%)	65 (76.5%)	13 (92.9%)		150 (79.4%)	35 (68.6%)	
Adverse	17 (5.0%)	4 (4.7%)	0		9 (4.8%)	4 (7.8%)	
Conditioning regimen, *n* (%)							
MA	339 (100%)	85 (100%)	14 (100%)		189 (100%)	51 (100%)	
HLA-A-, B-, and DR-mismatched grafts, *n* (%)							0.245
0	101 (29.8%)	85 (100%)	14 (100%)		1 (0.5%)	1 (2.0%)	
1	14 (4.1%)	0	0		13 (6.9%)	1 (2.0%)	
2	55 (16.2%)	0	0		40 (21.2%)	15 (29.4%)	
3	126 (49.9%)	0	0		135 (71.4%)	34 (66.7%)	
Donor-recipient sex-matched grafts, *n* (%)				0.249			0.481
Male–male	104 (30.7%)	22 (25.9%)	4 (28.6%)		62 (32.8%)	16 (31.4%)	
Male–female	86 (25.3%)	25 (29.4%)	1 (7.1%)		55 (29.1%)	14 (27.5%)	
Female–male	95 (28.0%)	32 (37.6%)	8 (57.1%)		44 (23.3%)	8 (17.6%)	
Female–female	47 (13.9%)	6 (7.1%)	1 (7.1%)		28 (14.8%)	12 (23.5%)	
Donor-recipient relationship, *n* (%)							0.439
Parent–child	128 (37.8%)	0	0		104 (55.0%)	24 (47.1%)	
Sibling–sibling	176 (51.9%)	85 (100%)	14 (100%)		58 (30.7%)	19 (37.3%)	
Child–parent	32 (9.4%)	0	0		24 (12.7%)	8 (15.7%)	
Other	3 (0.9%)	0	0		3 (1.6%)	0 (0%)	
ABO matched grafts, *n* (%)							0.087
Matched	201 (59.3%)	53 (62.4%)	9 (64.3%)	0.345	104 (55.0%)	35 (68.6%)	
Major mismatch	62 (18.3%)	19 (22.4%)	1 (7.1%)		39 (20.6%)	3 (5.9%)	
Minor mismatch	58 (17.1%)	9 (10.6%)	2 (14.3%)		36 (19.0%)	11 (21.6%)	
Bi-directional mismatch	18 (5.3%)	4 (4.7%)	2 (14.3%)		10 (5.3%)	2 (3.9%)	
EBMT score, *n* (%)				0.063			0.850
0	0	0	0		0	0	
1	73 (21.5%)	23 (27.1%)	3 (21.4%)		38 (20.1%)	9 (17.6%)	
2	156 (46.0%)	50 (58.8%)	7 (50.0%)		78 (41.3%)	21 (41.2%)	
3	76 (22.4%)	11 (12.9%)	1 (7.1%)		50 (26.5%)	14 (27.5%)	
4	25 (7.4%)	1 (1.2%)	2 (14.2%)		18 (9.5%)	4 (7.8%)	
5	9 (2.7%)	0	1 (7.1%)		5 (2.6%)	3 (5.9%)	
Cell compositions in allografts							
Infused nuclear cells, (range) 10^8^/kg	7.51 (3.98–16.77)	7.51 (3.98–14.75)(5.18–14.93)	7.12 (5.78–12.75)	0.896	7.40 (4.32–16.77)	7.69 (5.4–14.07)	0.262
Infused CD34^+^ cells, (range) 10^6^/kg	2.32 (0.50–9.78)	2.26 (0.76–9.78)	2.05 (1.16–5.04)	0.670	2.32 (0.50–9.47)	2.46 (1.04–8.80)	0.448
DLI after transplant, *n* (%)							
For relapse prophylaxis and intervention	28 (8.3%)	6 (7.1%)	4 (28.6%)	0.046	13 (6.9%)	5 (9.8%)	0.686
For relapse treatment	19 (5.6%)	2 (2.4%)	2 (14.3%)	0.171	12 (6.3%)	3 (5.9%)	0.902

*Abbreviations: HLA* human leukocyte antigen, *MSDT* HLA-matched sibling donor transplantation, *HBMT* unmanipulated haploidentical blood and marrow transplantation, *MRD* minimal residual disease, *neg* negative, *pos* positive, *AML* acute myeloid leukemia, *CR* complete remission, *MA* myeloablative regimen, *EBMT* European Group for Blood and Marrow Transplantation, *DLI* donor lymphocyte infusions

## Results

### Patient characteristics and transplant outcomes

Three hundred and thirty-nine patients and 340 cases were included in the retrospective and prospective study, respectively Fig. 1. All patients had less than 5% bone marrow blasts and met the morphological criteria for a leukemia-free state and complete remission. Table 1 and Additional file 1: Table S1 summarize the characteristics of these patients. A total of 87 patients received DLI, which was given for relapse prophylaxis (*n* = 10), intervention (*n* = 46), treatment (*n* = 28), or poor graft function (*n* = 3). The median dose of infused mononuclear cells was 1.0 × 10^8^/kg (range, 1.0 × 10^8^/kg to 2.99 × 10^8^/kg). There were no significant differences in the percentages of patients who received DLI for relapse prophylaxis and intervention among the pre-MRD-positive subgroups in both the retrospective study and the prospective study (Tables 1 and 2).

**Table 2:** Patient and donor characteristics in the prospective study

Characteristics	All patients	MSDT			HBMT		
		MRDneg	MRDpos		MRDneg	MRDpos	
Number of patients	340	62	20		202	56	
Median age (range), years	32 (3–65)	39 (4–55)	41.5 (7–62)	0.612	30 (3–65)	26 (4–61)	0.151
Weight (range), kg	62 (15.5–140)	63 (15.5–91)	65.25 (28–95)	0.631	62 (17–140)	58 (19–92)	0.090
Male, *n* (%)	208 (61.2%)	32 (51.6%)	15 (75.0%)	0.066	127 (62.9%)	34 (60.7%)	0.768
Diagnosis, n (%)				0.146			0.603
De novo AML	318 (93.5%)	61 (98.4%)	18 (90.0%)		187 (92.6%)	52 (92.9%)	
Secondary AML	22 (6.5%)	1 (1.6%)	2 (10.0%)		15 (7.4%)	4 (7.1%)	
Disease status, *n* (%)							0.165
CR1	293 (86.2%)	56 (90.3%)	15 (75.0%)	0.080	177 (87.6%)	45 (80.4%)	
CR > 1	47 (13.8%)	6 (9.7%)	5 (25.0%)		25 (12.4%)	11 (19.6%)	
FLT3-ITD mutation				0.390			0.610
Yes	49 (14.4%)	11 (17.7%)	2 (10.0%)		27 (13.4%)	9 (16.1%)	
No	291 (85.6%)	51 (82.3%)	18 (90.0%)		175 (86.6%)	47 (83.9%)	
Cytogenetics				0.500			0.277
Favorable	43 (12.6%)	7 (11.3%)	1 (5.0%)		28 (13.9%)	7 (12.5%)	
Intermediate	263 (77.4%)	50 (80.6%)	16 (80.0%)		157 (77.7%)	40 (71.4%)	
Adverse	34 (10.0%)	5 (8.1%)	3 (15.0%)		17 (8.4%)	9 (16.1%)	
Conditioning regimen, *n* (%)							
MA	340 (100%)	62 (100%)	20 (100%)		202 (100%)	56 (100%)	
HLA-A-, B-, and DR-mismatched grafts, *n* (%)							0.599
0	84 (24.7%)	62 (100%)	20 (100%)		2 (1.0%)	0	
1	7 (2.1%)	0	0		5 (2.5%)	2 (3.6%)	
2	31 (9.1%)	0	0		22 (10.9%)	9 (16.1%)	
3	218 (64.1%)	0	0		173 (85.6%)	45 (80.4%)	
Donor-recipient sex-matched grafts, *n* (%)							
Male–male	136 (40.0%)	15 (24.2%)	6 (30.0%)		94 (46.5%)	21 (37.5%)	
Male–female	86 (25.3%)	14 (22.6%)	3 (15.0%)		55 (27.2%)	14 (25.0%)	
Female–male	75 (22.1%)	18 (29.0%)	2 (10.0%)		33 (16.3%)	15 (26.8%)	
Female–female	43 (12.6%)	15 (24.2%)	9 (45.0%)		20 (9.9%)	6 (10.7%)	
Donor-recipient relationship, *n* (%)							0.283
Parent–child	132 (38.8%)	0	0		99 (49.0%)	33 (58.9%)	
Sibling–sibling	153 (45.0%)	62 (100%)	20 (100%)		61 (30.2%)	10 (17.9%)	
Child–parent	49 (14.4%)	0	0		37 (18.3%)	12 (21.4%)	
Other	6 (1.8%)	0	0		5 (2.5%)	1 (1.8%)	
ABO matched grafts, *n* (%)							0.344
Matched	179 (52.6%)	40 (64.5%)	14 (70.0%)	0.660	97 (48.0%)	28 (50.0%)	
Major mismatch	70 (20.6%)	10 (16.1%)	4 (20.0%)		45 (22.3%)	11 (19.6%)	
Minor mismatch	72 (21.2%)	8 (12.9%)	2 (10.0%)		46 (22.8%)	16 (28.6%)	
Bi-directional mismatch	19 (5.6%)	4 (6.5%)	0		14 (6.9%)	1 (1.8%)	
EBMT score, *n* (%)				0.125			0.546
0	2 (0.6%)	1 (1.6%)	1 (5.0%)		0	0	
1	66 (19.4%)	21 (33.9%)	6 (30.0%)		31 (15.3%)	8 (14.3%)	
2	145 (42.6%)	28 (45.2%)	6 (30.0%)		90 (44.6%)	21 (37.5%)	
3	91 (26.8%)	11 (17.7%)	4 (20.0%)		58 (28.7%)	18 (32.1%)	
4	30 (8.8%)	1 (1.6%)	3 (15.0%)		20 (9.9%)	6 (10.7%)	
5	6 (1.8%)	0	0		3 (1.5%)	3 (5.4%)	
Cell compositions in allografts							
Infused nuclear cells, (range) 10^8^/kg	7.83 (2.27–16.66)	7.59 (5.18–14.93) (5.18–14.93)	7.22 (2.27–9.29)	0.201	7.88 (3.93–15.97)	8.17 (3.44–16.66)	0.517
Infused CD34^+^ cells, (range) 10^6^/kg	2.55 (0.22–10.95)	2.53 (0.41–6.43)	2.65 (0.90–5.47)	0.829	2.49 (0.22–10.95)	2.78 (0.38–7.20)	0.340
DLI after transplant, *n* (%)							
For relapse prophylaxis and intervention	32 (9.4%)	5 (8.1%)	4 (20.0%)	0.211	8 (4.0%)	14 (25.0%)	<0.001
For relapse treatment	8 (2.4%)	2 (3.2%)	2 (10.0%)	0.249	3 (1.5%)	2 (3.6%)	0.297

*Abbreviations: HLA* human leukocyte antigen, *MSDT* HLA-matched sibling donor transplantation, *HBMT* unmanipulated haploidentical blood and marrow transplantation, *MRD* minimal residual disease, *neg* negative, *pos* positive, *AML* acute myeloid leukemia, *CR* complete remission, *MA* myeloablative regimen, *EBMT* European Group for Blood and Marrow Transplantation, *DLI* donor lymphocyte infusions

All except for one patient (338; 99.7%) in the retrospective group achieved sustained, full-donor chimerism. The cumulative, 100-day incidence of acute GVHD grades II to IV for pre-MRDpos patients who underwent MSDT was significantly lower than those treated with haplo-SCT (7 vs. 43%, *P* = 0.042) (Table 3). The cumulative incidences of acute GVHD grades III to IV for patients who underwent MSDT and those treated with haplo-SCT were comparable (7 vs. 3%, *P* = 0.173). The 4-year cumulative incidence of severe chronic GVHD was comparable between patients who underwent MSDT and those treated with haplo-SCT (10 vs. 10%, *P* = 0.841) in the retrospective group. After a median follow-up of 1216 days (range, 758–1700 days) for live cases, the 4-year cumulative incidences of non-relapse mortality and relapse were 13 and 16%, respectively. The 4-year probabilities of LFS and OS were 71 and 74%, respectively (Table 3).

**Table 3:** Transplant outcomes for patients that underwent allogeneic stem cell transplantation in the retrospective and prospective study

		Neutrophil engraftment	Platelet engraftment	Grades 2–4 acute GVHD	Chronic GVHD at 4 years	Relapse at 4 years	NRM at 4 years	LFS at 4 years	OS at 4 years
Retrospective study group (*n* = 339)									
MSDT (*n* = 99)	MRDneg (group A)	98% (95% CI, 96–100%)	98% (95% CI, 94–100%)	9% (95% CI, 3 to 15%) ^‡, ##^	58% (95% CI, 45 to 71%)	11% (95% CI, 4 to 18%)	16% (95% CI, 7 to 25%)	73% (95% CI, 63 to 83%)	76% (95% CI, 66 to 86%)
	MRDpos (group B)	93% (95% CI, 79–100%) ^£^	93% (95% CI, 79–100%)	7% (95% CI, 0 to 21%) ^†, #^	66% (95% CI, 35 to 97%)	60% (95% CI, 22 to 98%)	7% (95% CI, 0 to 21%)	33% (95% CI, 2 to 64%)	33% (95% CI, 2 to 64%)
Haplo-SCT (*n* = 240)	MRDneg (group C)	99% (95% CI, 99–100%)	99% (95% CI, 97–100%)	36% (95% CI, 29 to 43%)	48% (95% CI, 40 to 56%)	15% (95% CI, 10 to 20%)	14% (95% CI, 9 to 19%)	71% (95% CI, 65 to 77%)	75% (95% CI, 69 to 81%)
	MRDpos (group D)	98% (95% CI, 96–100%)	97% (95% CI, 93–100%)	43% (95% CI, 29 to 57%)	70% (95% CI, 56 to 84%) *	19% (95% CI, 5 to 33%)	8% (95% CI, 1 to 15%)	73% (95% CI, 58 to 88%)	75% (95% CI, 60 to 90%)
Prospective study group (*n* = 340) ^a^									
MSDT (*n* = 82)	MRDneg (group E)	98% (95% CI, 95–100%)	98% (95% CI, 94–100%)	10% (95% CI, 2 to 17%) ^$, $$^	56% (95% CI, 39 to 72%)	7% (95% CI, 0 to 13%)	5% (95% CI, 0 to 11%)	88% (95% CI, 79 to 97%)	94% (95% CI, 87 to 100%)
	MRDpos (group F)	95% (95% CI, 85–100%) ^££^	95% (95% CI, 85–100%)	5% (95% CI, 0 to 15%) ^††, ‡‡^	41% (95% CI, 20 to 62%)	36% (95% CI, 14 to 58%)	16% (95% CI, 0 to 33%)	48% (95% CI, 25 to 71%)	64% (95% CI, 42 to 86%)
Haplo-SCT (*n* = 258)	MRDneg (group G)	99% (95% CI, 99–100%)	99% (95% CI, 99–100%)	28% (95% CI, 21 to 35%)	40% (95% CI, 31 to 49%)	7% (95% CI, 3 to 11%)	18% (95% CI, 12 to 24%)	75% (95% CI, 69 to 81%)	78% (95% CI, 72 to 84%)
	MRDpos (group H)	97% (95% CI, 91–100%)	98% (95% CI, 94–100%)	32% (95% CI, 20 to 44%)	73% (95% CI, 52 to 94%) **	13% (95% CI, 4 to 22%)	7% (95% CI, 0 to 14%)	80% (95% CI, 69 to 91%)	83% (95% CI, 73 to 93%)

The differences in any of the transplant outcomes between the four groups were analyzed with a log-rank test

*Abbreviations*: *GVHD* graft-versus-host disease, *NRM* non-relapse mortality, *LFS* leukemia-free survival, *OS* overall survival, *MRD* minimal residual disease, *MSDT* human leukocyte antigen-matched sibling donor transplantation, *MRDpos* MRD positive, *MRDneg* MRD negative, *Haplo-SCT* haploidentical stem cell transplantation

^£^ *P* < 0.01 compared with group D

^££^ *P* < 0.05 compared with group D

^‡^ *P* < 0.05 compared with group C

^##^ *P* < 0.01 compared with group D

^†^ *P* < 0.05 compared with group C

^#^ *P* < 0.01 compared with group D

*P = 0.980 compared with group B

^$^ *P* < 0.01 compared with group G

^$$^ *P* < 0.01 compared with group H

^††^ *P* < 0.05 compared with group G

^‡‡^ *P* < 0.05 compared with group H

**P = 0.223 compared with group F

^a^Indicates the transplant outcomes of patients in the prospective study are listed as chronic GVHD, relapse, NRM, LFS and OS at 2 years

All patients (340; 100%) in the prospective group achieved sustained, full-donor chimerism. The cumulative, 100-day incidence of acute GVHD grades II to IV for pre-MRDpos patients who underwent MSDT was significantly lower than those treated with haplo-SCT (5 vs. 32%, *P* = 0.019) (Table 3). The cumulative incidences of acute GVHD grades III to IV for patients who underwent MSDT and those receiving haploidentical allografts were comparable (5 vs. 4%, *P* = 0.501). The 4-year cumulative incidence of severe chronic GVHD was comparable between patients who underwent MSDT and those receiving haploidentical allografts (8 vs. 5%, *P* = 0.386) in the prospective group. After a median follow-up of 400 days (range, 32–756 days), the 2-year cumulative incidences of non-relapse mortality and relapse were 14 and 9%, respectively. The 2-year probabilities of LFS and OS were 77 and 81%, respectively (Table 3).

### Impact of pre-MRD on outcomes in patients receiving haplo-SCT versus MSDT

In the retrospective group, patients undergoing haplo-SCT were classified as being in the pre-MRDneg group (*n* = 189) or pre-MRDpos group (*n* = 51, Table 1). Pre-MRDneg and pre-MRDpos patients had comparable incidences of relapse (15 vs. 19%, *P* = 0.866) and NRM (14 vs. 8%, *P* = 0.287) and similar probabilities of LFS (71 vs. 73%, *P* = 0.567) and OS (76 vs. 75%, *P* = 0.717) (Table 3 and Additional file 2: Figure S1 A–D). Multivariate analysis showed that there were no associations of pre-MRDpos status with relapse, NRM, LFS, or OS. Patients undergoing MSDT were also classified as being in the pre-MRDneg group (*n* = 85) or pre-MRDpos group (*n* = 14, Table 1). Compared to pre-MRDpos patients, pre-MRDneg patients had lower incidences of relapse (11 vs. 60%, *P* < 0.001), similar incidences of NRM (16 vs. 7%, *P* = 0.743), and higher probabilities of LFS (73 vs. 33%, *P* = 0.001) and OS (76 vs. 33%, *P* = 0.001) (Table 3 and Additional file 3: Figure S2 A–D). Multivariate analysis showed that pre-MRDpos status was associated with leukemia relapse (HR, 8.860; 95% CI, 3.173–24.739; *P* < 0.001), LFS (HR, 5.482; 95% CI, 2.306–13.033; *P* < 0.001), and OS (HR, 5.700; 95% CI, 2.327–13.962; *P* < 0.001).

In the prospective group, patients undergoing haplo-SCT were classified as being in the pre-MRDneg group (*n* = 202) or pre-MRDpos group (*n* = 56, Table 2). Pre-MRDneg and pre-MRDpos patients had comparable incidences of relapse (7 vs. 13%, *P* = 0.161) and NRM (18 vs. 7%, *P* = 0.083) and similar probabilities of LFS (75 vs. 80%, *P* = 0.583) and OS (78 vs. 83%, *P* = 0.516) (Table 3 and Additional file 4: Figure S3 A–D). Multivariate analysis showed that pre-MRDpos status was not associated with NRM, leukemia relapse, LFS, or OS. Patients undergoing MSDT were also classified as being in the pre-MRDneg group (*n* = 62) or pre-MRDpos group (*n* = 20, Table 2). Compared to pre-MRDpos patients, pre-MRDneg patients had lower incidences of relapse (7 vs. 36%, *P* < 0.001) and NRM (5 vs. 16%, *P* = 0.033) and higher probabilities of LFS (88 vs. 48%, *P* < 0.001) and OS (94 vs. 64%, *P* < 0.001) (Table 3 and Additional file 5: Figure S4 A–D). Multivariate analysis showed that pre-MRDpos status was associated with leukemia relapse (HR, 8.331; 95% CI, 2.395–28.893; *P* = 0.001), LFS (HR, 5.821; 95% CI, 2.209–15.338; *P* < 0.001), and OS (HR, 8.732; 95% CI, 2.254–33.819; *P* = 0.002). These results from the retrospective and prospective analysis suggest that haplo-SCT may have better anti-leukemia effects in MSDT in eradicating pre-MRD.

### Haplo-SCT achieved better outcomes than MSDT for patients with pre-MRD-positive AML

There were 65 pre-MRD-positive (pre-MRDpos) patients in the retrospective group (Tables 1 and 3). Compared to those with pre-MRDpos receiving haplo-SCT, patients with pre-MRDpos who underwent MSDT had a higher incidence of relapse (57 vs. 19%, *P* < 0.001) and lower probabilities of LFS (29 vs. 73%, *P* < 0.001) and OS (33 vs. 75%, *P* = 0.001), whereas there was no statistically difference in NRM (14 vs. 8%, *P* = 0.318; Additional file 6: Figure S5 A–D). Multivariate analysis showed that haplo-SCT was associated with a low incidence of leukemia relapse (*P* = 0.010) and with better LFS (*P* = 0.041) and OS (*P* = 0.007) (Additional file 1: Table S1). In the prospective group, there were 76 pre-MRDpos patients (Tables 2 and 3). Compared to those with pre-MRDpos receiving haplo-SCT, patients with pre-MRDpos who underwent MSDT had a higher incidence of relapse (36 vs. 13%, *P* = 0.017) and lower probabilities of LFS (48 vs. 80%, *P* = 0.007) and a lower probabilities of OS (64% vs. 83%, *P* = 0.062) trend, whereas there was no statistical difference in NRM (16 vs. 7%, *P* = 0.247; Additional file 7: Figure S6 A–D). Multivariate analysis showed that haplo-SCT was associated with a low incidence of leukemia relapse (*P* = 0.002) and with better LFS (*P* = 0.002) and OS (*P* = 0.040) (Additional file 8: Table S2).

**Fig. 2:**
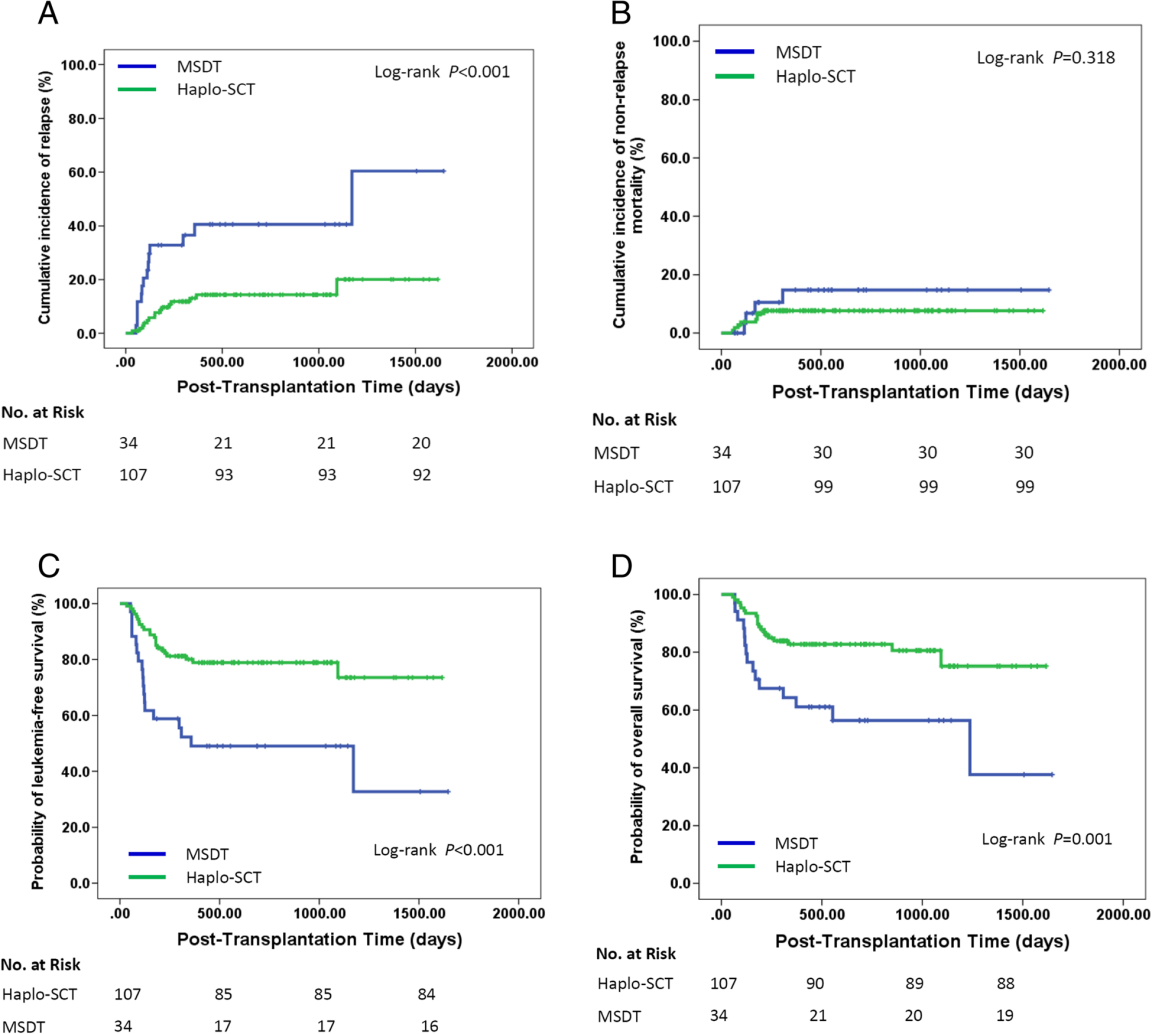
Relationship between transplant modality and transplant outcomes for AML patients with pre-transplantation MRD who underwent allo-SCT (n = 141). Kaplan–Meier estimates of (**a**) cumulative incidence of relapse mortality, (**b**) cumulative incidence of non-relapse, (**c**) leukemia-free survival, and (**d**) overall survival

After combination of pre-MRDpos cases in the retrospective group and the prospective group (*n* = 141), compared to those with pre-MRDpos receiving haplo-SCT (*n* = 107), patients with pre-MRDpos who underwent MSDT (*n* = 34) had a higher incidence of relapse (55 vs. 19%, *P* < 0.001) and lower probabilities of LFS (33 vs. 74%, *P* < 0.001) and OS (38 vs. 83%, *P* = 0.001), whereas there was no statistical difference in NRM (12 vs. 7%, *P* = 0.318; Fig. 2a–d). Multivariate analysis showed that haplo-SCT was associated with a low incidence of leukemia relapse (HR, 0.360; 95% CI, 0.159–0.813; *P* = 0.014) and with better LFS (HR, 0.334; 95% CI, 0.165–0.677; *P* = 0.001) and OS (HR, 0.340; 95% CI, 0.155–0.743; *P* = 0.007) (Table 4).

**Table 4:** Multivariate analysis of factors associated with outcomes of patients with pre-transplantation MRD who underwent allo-SCT both in the retrospective study and the prospective study (*n* = 141)

Covariate	Univariate analysis			Multivariate analysis		
	HR	95% CI	*P* value	HR	95% CI	*P* value
Relapse						
Disease status (CR1 vs. CR > 1)	4.736	2.113–10.617	<0.001	5.852	2.569–13.652	<0.001
Transplant modality	0.318	0.142–0.712	0.005	0.360	0.159–0.813	0.014
Chronic GVHD (yes vs. no)	0.834	0.712–0.977	0.024	0.793	0.669–0.939	0.007
FLT3-ITD (yes vs. no)	2.710	0.914–8.041	0.072			
Transplant-related mortality						
Recipient age	1.051	0.998–1.108	0.061			
Neutrophil engraftment	1.213	1.019–1.445	0.030	1.213	1.019–1.445	0.030
Leukemia-free survival						
Disease status (CR1 vs. CR > 1)	3.542	1.715–7.318	0.001	4.554	2.127–9.752	<0.001
Transplant modality	0.300	0.149–0.602	0.001	0.334	0.165–0.677	0.001
Chronic GVHD (yes vs. no)	0.812	0.705–0.934	0.004	0.783	0.675–0.909	0.001
FLT3-ITD (yes vs. no)	2.501	0.951–6.575	0.063			
Overall survival						
Disease status (CR1 vs. CR > 1)	2.634	1.171–5.923	0.019	2.269	1.002–5.137	0.049
Transplant modality	0.309	0.143–0.670	0.003	0.340	0.155–0.743	0.007

All variables were first included in the univariate analysis; only variables with *P* < 0.1 were included in the Cox proportional hazards model with time-dependent variables

*Abbreviations: MSDT* human leukocyte antigen-matched sibling donor transplantation, *HR* hazard ratio, *CI* confidence interval, *EBMT* European Group for Blood and Marrow Transplantation

Considering the effects of pre-MRD on relapse after transplantation were different according to the level of leukemic cells [16]. Total pre-MRDpos patients (*n* = 141) were categorized into the following two groups: group A = patients with a detectable MRD load less than the quantitative range (<10^−2^ leukemic cells; *n* = 86) and group B = patients with MRD load between ≥10^−2^ leukemic cells (*n* = 55). For cases in group A, the cumulative incidence of relapse and NRM was (54 vs. 11%, *P* = 0.004) and (% vs. 8%, *P* = 0.634), respectively, after HLA-matched allografts and haplo-SCT. The probability of LFS and OS was (41 vs. 81%, *P* = 0.019) and (44 vs. 83%, *P* = 0.027), respectively, after HLA-matched allografts and haplo-SCT. For cases in group B, the cumulative incidence of relapse and NRM was (48 vs. 36%, *P* = 0.029) and (23 vs. 7%, *P* = 0.118), respectively, after HLA-matched allografts and haplo-SCT. The probability of LFS and OS was (29 vs. 57%, *P* = 0.008) and (37 vs. 59%, *P* = 0.020), respectively, after HLA-matched allografts and haplo-SCT (Additional file 9: Figure S7 A–D). Multivariate analysis also demonstrated that haplo-SCT was associated with leukemia relapse, LFS, and OS, after classification of the pre-MRDpos cases into four groups according to MRD load and transplant modalities (Additional file 10: Table S3).

After excluding the cases who received DLI from the pre-MRDpos patients, 105 subjects remain. In this subgroup (*n* = 105), compared to those treated with haplo-SCT (*n* = 83), patients who underwent MSDT (*n* = 22) had a higher incidence of relapse (45 vs. 5%, *P* = 0.001) and lower probabilities of LFS (45 vs. 88%, *P* = 0.006) and OS (48 vs. 88%, *P* = 0.027), whereas there was no statistically difference in NRM (10 vs. 7%, *P* = 0.683; Additional file 11: Figure S8 A–D). Multivariate analysis demonstrated that haplo-SCT was associated with leukemia relapse, LFS, and OS in this subgroup of patients (Additional file 12: Table S4).

## Discussion

The most interesting finding of the present study is that pre-MRD, as determined by MFC, showed no association with increased risk of relapse in patients who underwent haplo-SCT after the retrospective and prospective analysis. This contrasts with the results observed in MSDT settings, which show a negative effect of pre-MRD on relapse [12, 14, 15, 17, 20]. Subgroup analysis that only included pre-MRD-positive patients with AML also showed that cases undergoing unmanipulated haplo-SCT had a lower incidence of relapse compared to those who received MDST. Our results suggest that unmanipulated haplo-SCT may be better than MSDT in eradicating pre-MRD.

Several studies have demonstrated the negative effects of pre-MRD on outcomes after MSDT [12, 14, 15, 17, 20]. A retrospective study by Walter et al. [18] investigated 100 cases with AML undergoing myeloablative SCT from HLA-matched related or unrelated donors and found that the 2-year estimates of relapse were 64.9 and 17.6% for MRD-positive and MRD-negative patients, respectively. Another study of 152 AML patients reported that the 1-year relapse incidence was higher in patients with pre-MRD than without pre-MRD (32.6 vs. 14.4%, *P* = 0.002) [17]. In our study, we found compelling evidence that pre-MRD had negative effects on AML relapse in the MSDT setting. These data indicate that treating AML with MSDT or MUDT could not overcome the negative effects of pre-MRD on transplant outcomes.

Importantly, for the first time, we observed that there were no negative effects of pre-SCT MRD on relapse following the unmanipulated haplo-SCT modality based on the retrospective and prospective analysis (Tables 3, and 4). Further analysis indicated that haplo-SCT was also associated with lower incidence of relapse and better survival after classification of pre-MRDpos cases into two groups according to the level of leukemic cells. Relapse is affected by several factors, such as the conditioning regimen, DLI, and disease status [2, 23, 25]. In this study, the difference in the conditioning regimen between haplo-SCT and MSDT is that ATG was used only in the haploidentical setting. Although an in vitro experiment demonstrated that ATG at clinically relevant concentrations can kill leukemic blasts [33], ATG does not seem to play a role in decreasing the incidence of leukemia relapse in either the MSDT or the MUDT setting [5, 6]. Notably, chronic GVHD induces GVL effects after unmanipulated haplo-SCT for AML [34]. In addition, ATG may decrease the incidence of cGVHD [5, 6]. Therefore, the lower incidence of relapse in pre-MRD-positive patients with AML after haplo-SCT versus after MSDT cannot be explained by the use of ATG.

DLI is an effective strategy for prophylaxis and for intervention of leukemia relapse in MSDT, MUDT, and haplo-SCT settings [24, 25]. Our previous study demonstrated that DLI could overcome the negative effects of MRD on transplant outcomes [25]. In the present study, the percentages of pre-MRD-positive patients who received DLI for relapse prophylaxis and intervention were similar in the haplo-SCT group and the MSDT group. Furthermore, after excluding the cases who received DLI from the pre-MRDpos patients, we found that haplo-SCT was also associated with lower incidence of leukemia relapse and superior survival (Additional file 7: Figure S6 and Additional file 1: Table S1). Thus, the superior effects of unmanipulated haplo-SCT in eradicating pre-SCT MRD prior to MSDT could not be ascribed to the effects of DLI on leukemia relapse [25].

In this study, the similar patient characteristics, such as diagnosis and disease status, along with the evidence that haplo-SCT but not MSDT significantly decreased the percentage of patients with positive MRD, further support the idea that allografts from haploidentical donors may have strong anti-leukemia effects, given the negative effects of post-SCT MRD on relapse that have been reported by others [14, 15, 35] and that were observed in our study. In fact, Mo et al. [36] found that for AML patients, the outcomes were comparable in cases that were resistant to the first course of induction chemotherapy (IC1st-resistant) and in IC1st-sensitive cases, which suggests that unmanipulated haplo-SCT can mitigate the poor outcomes of AML that is resistant to the first course of induction chemotherapy. cGVHD was associated with anti-leukemia effects, and the fact that haplo-SCT has a high incidence of cGVHD compared to MSDT, also no significance was demonstrated, may contribute to the strong anti-leukemia effects, as previously described by Mo et al. [34] Due to the better GVL effects of haplo-SCT, along with comparable NRM between haplo-SCT and MSDT, patients with positive pre-SCT MRD receiving allografts from haploidentical donor experienced superior LFS and OS. Therefore, our results not only suggest strong anti-leukemia effects, they also indicate the superiority of eradicating pre-SCT MRD of haploidentical allografts. A multicenter, clinical trial is needed to confirm our findings both in the setting of unmanipulated haplo-SCT modality with ATG-based treatment [10, 23] and in other haplo-SCT modalities, such as unmanipulated haplo-SCT with PT/Cy [5].

In a recent study, Milano et al. [11] reported that treating pre-MRDpos patients with CBT led to a higher rate of survival and a lower rate of relapse than those of a transplant from an HLA-mismatched unrelated donor. The authors found similar survival rate between CBT and MUDT, although the risk of relapse was higher after receipt of a transplant from an MUD than after receipt of a transplant from a cord-blood donor [11]. The results provided by Milano et al. [11] and us suggest that a study comparing the differences in the effects between haplo-SCT and CBT on clinical outcomes of cases with pre-MRDpos is warranted.

## Conclusion

In conclusion, our results, for the first time, indicated that haplo-SCT had a stronger effect than MSDT on the eradication of pre-MRD in patients with AML based on the retrospective and prospective analysis, which suggests the GVL effects of unmanipulated haplo-SCT. Therefore, this report provides the first evidence that, for pre-MRD-positive AML patients, unmanipulated haplo-SCT should be preferred over MSDT for eradicating leukemia cells, particularly for patients without HLA-identical sibling donors.

## Additional files

Additional file 1: Table S1. Multivariate analysis of factors associated with outcomes of patients with pre-transplantation MRD-positive who underwent allo-SCT in the retrospective study (*n* = 65). (DOCX 18 kb)

Additional file 2: Figure S1. Relationship between pre-stem cell transplantation minimal residual disease (pre-SCT MRD), as determined by multiparameter flow cytometry, and transplant outcomes for acute myeloid leukemia patients (*n* = 240) who underwent haploidentical stem cell transplantation in the retrospective study. Estimates of (A) cumulative incidence of non-relapse mortality, (B) cumulative incidence of relapse, (C) leukemia-free survival, and overall survival. MRDneg = negative MRD status; MRDpos = positive MRD status. (DOCX 406 kb)

Additional file 3: Figure S2. Relationship between pre-stem cell transplantation minimal residual disease (pre-SCT MRD), as determined by multiparameter flow cytometry, and transplant outcomes for acute myeloid leukemia patients (*n* = 99) who underwent HLA-matched sibling donor transplantation in the retrospective study. Estimates of (A) cumulative incidence of non-relapse mortality, (B) cumulative incidence of relapse, (C) leukemia-free survival, and overall survival. MRDneg = negative MRD status; MRDpos = positive MRD status. (DOCX 396 kb)

Additional file 4: Figure S3. Relationship between pre-stem cell transplantation minimal residual disease (pre-SCT MRD), as determined by multiparameter flow cytometry, and transplant outcomes for acute myeloid leukemia patients (*n* = 258) who underwent haploidentical stem cell transplantation in the prospective study. Estimates of (A) cumulative incidence of non-relapse mortality, (B) cumulative incidence of relapse, (C) leukemia-free survival, and overall survival. MRDneg = negative MRD status; MRDpos = positive MRD status. (DOCX 275 kb)

Additional file 5: Figure S4. Relationship between pre-stem cell transplantation minimal residual disease (pre-SCT MRD), as determined by multiparameter flow cytometry, and transplant outcomes for acute myeloid leukemia patients (*n* = 82) who underwent HLA-matched sibling donor transplantation in the prospective study. Estimates of (A) cumulative incidence of non-relapse mortality, (B) cumulative incidence of relapse, (C) leukemia-free survival, and overall survival. MRDneg = negative MRD status; MRDpos = positive MRD status. (DOCX 265 kb)

Additional file 6: Figure S5. Relationship between transplant modality and transplant outcomes for AML patients with pre-transplantation MRD who underwent allo-SCT (*n* = 65). Estimates of (A) cumulative incidence of non-relapse mortality, (B) cumulative incidence of relapse, (C) leukemia-free survival, and overall survival. MRDneg = negative MRD status; MRDpos = positive MRD status. (DOCX 395 kb)

Additional file 7: Figure S6. Relationship between transplant modality and transplant outcomes for AML patients with pre-transplantation MRD who underwent allo-SCT (*n* = 76), who underwent haploidentical stem cell transplantation. Estimates of (A) cumulative incidence of non-relapse mortality, (B) cumulative incidence of relapse, (C) leukemia-free survival, and overall survival. MRDneg = negative MRD status; MRDpos = positive MRD status. (DOCX 340 kb)

Additional file 8: Table S2. Multivariate analysis of factors associated with outcomes of patients with pre-transplantation MRD-positive who underwent allo-SCT in the prospective study (*n* = 76). (DOCX 19 kb)

Additional file 9: Figure S7. Association between transplant modalities and outcomes for AML patients with pre-transplantation MRD who underwent allo-SCT, stratified by the level of leukemic cells. Estimates of (A) cumulative incidence of non-relapse mortality, (B) cumulative incidence of relapse, (C) leukemia-free survival, and overall survival. (DOCX 520 kb)

Additional file 10: Table S3. Multivariate analysis of factors associated with outcomes of patients with pre-transplantation MRD-positive who underwent allo-SCT both in the retrospective study and the prospective study categorization in two groups according to MRD load and transplant modalities (*n* = 141). (DOCX 21 kb)

Additional file 11: Figure S8. Relationship between transplant modality and transplant outcomes for AML patients with pre-transplantation MRD who underwent allo-SCT excluding the cases who received DLI (*n* = 105). Estimates of (A) cumulative incidence of non-relapse mortality, (B) cumulative incidence of relapse, (C) leukemia-free survival, and overall survival. (DOCX 394 kb)

Additional file 12: Table S4. Multivariate analysis of factors associated with outcomes of patients with pre-transplantation MRD-positive who underwent allo-SCT both in the retrospective study and the prospective study without receiving donor lymphocyte infusion (*n* = 105). (DOCX 19 kb)

## Abbreviations

AML: Acute myeloid leukemia
ATG: Anti-thymocyte globulin
CBT: Cord blood transplantation
CI: Confidence interval
CR: Complete remission
DLI: Donor lymphocyte infusion
EBMT: European Group for Blood and Marrow Transplantation
FCM: Flow cytometry
GF: Graft failure
GVHD: Graft-versus-host disease
GVL: Graft-versus-leukemia
Haplo-SCT: Haploidentical stem cell transplantation
HR: Hazard ratio
LFS: Leukemia-free survival
MRD: Minimal residual disease
MSDT: Human leukocyte antigen (HLA)-matched sibling donor transplantation
MUDT: HLA-matched unrelated donor transplantation
neg: Negative
NRM: Non-relapse mortality
OS: Overall survival
pos: Positive
Pre-MRD: Pre-transplantation MRD
PT/CY: Post-cyclophosphamide
