# Supplementary material for: Haploidentical allograft is superior to matched sibling donor allograft in eradicating pre-transplantation minimal residual disease of AML patients as determined by multiparameter flow cytometry: a retrospective and prospective analysis
Source: J Hematol Oncol. 2017 Jul 4;10:134. doi: 10.1186/s13045-017-0502-3 (PMC5496245; doi:10.1186/s13045-017-0502-3)
Supplement: Supplementary file 1 — Multivariate analysis of factors associated with outcomes of patients with pre-transplantation MRD-positive who underwent allo-SCT in the retrospective study (n = 65). (DOCX 18 kb) [file 13045_2017_502_MOESM1_ESM.docx]

**Table S1**. Multivariate analysis of factors associated with outcomes of patients with pre-transplantation MRD positive who underwent allo-SCT in the retrospective study (n=65)

| Covariate | Univariate analysis | | |  | Multivariate analysis | | |
| --- | --- | --- | --- | --- | --- | --- | --- |
|  | HR | 95% CI | *P*-value |  | HR | 95% CI | *P*-value |
| Relapse |  |  |  |  |  |  |  |
| Disease status (CR1 vs. CR﹥1) | 2.729 | 0.926-8.044 | 0.069 |  |  |  |  |
| Transplant modality | 0.284 | 0.102-0.789 | 0.016 |  | 0.314 | 0.112-0.883 | 0.028 |
| Platelet engraftment | 0.095 | 0.011-0.819 | 0.032 |  |  |  |  |
| Chronic GVHD (yes vs. no) | 0.368 | 0.130-1.039 | 0.059 |  |  |  |  |
| FLT3-ITD (yes vs. no) | 8.063 | 2.149-30.255 | 0.002 |  | 7.030 | 1.790-27.611 | 0.005 |
| Leukemia-free survival |  |  |  |  |  |  |  |
| Neutrophil engraftment | 0.016 | 0.001-0.252 | 0.003 |  | 0.046 | 0.002-0.880 | 0.041 |
| Platelet engraftment | 0.051 | 0.010-0.268 | ﹤0.001 |  |  |  |  |
| Chronic GVHD (yes vs. no) | 0.236 | 0.090-0.616 | 0.003 |  | 0.213 | 0.077-0.594 | 0.003 |
| Disease status (CR1 vs. CR﹥1) | 2.467 | 0.933-6.526 | 0.069 |  | 3. 027 | 0.989-9.261 | 0.052 |
| Transplant modality | 0.375 | 0.152-0.924 | 0.033 |  |  |  |  |
| FLT3-ITD (yes vs. no) | 5.851 | 1.638-20.897 | 0.007 |  | 5.216 | 1.265-21.499 | 0.022 |
| Overall survival |  |  |  |  |  |  |  |
| Transplant modality | 0.329 | 0.132-0.823 | 0.017 |  | 0.265 | 0.102-0.691 | 0.007 |
| Platelet engraftment | 0.010 | 0.001-0.112 | ﹤0.001 |  | 0.006 | 0.001-0.073 | ﹤0.001 |
| FLT3-ITD (yes vs. no) | 8.080 | 2.263-28.848 | 0.001 |  |  |  |  |
| Disease status (CR1 vs. CR﹥1) | 2.540 | 0.962-6.703 | 0.060 |  |  |  |  |

**Abbreviations:** HR=hazard ratio; CI=confidence interval; MRD=minimal residual disease; allo-SCT=allogeneic stem cell transplantation

* All variables were first included in the univariate analysis; only variables with *P* < 0.1 were included in the Cox proportional hazards model with time-dependent variables.
