# Supplementary material for: Haploidentical allograft is superior to matched sibling donor allograft in eradicating pre-transplantation minimal residual disease of AML patients as determined by multiparameter flow cytometry: a retrospective and prospective analysis
Source: J Hematol Oncol. 2017 Jul 4;10:134. doi: 10.1186/s13045-017-0502-3 (PMC5496245; doi:10.1186/s13045-017-0502-3)
Supplement: Supplementary file 3 — Relationship between pre-stem cell transplantation minimal residual disease (pre-SCT MRD), as determined by multiparameter flow cytometry, and transplant outcomes for acute myeloid leukemia patients (n = 99) who underwent HLA-matched sibling donor transplantation in the retrospective study. Estimates of (A) cumulative incidence of non-relapse mortality, (B) cumulative incidence of relapse, (C) leukemia-free survival, and overall survival. MRDneg = negative MRD status; MRDpos = positive MRD status. (DOCX 396 kb) [file 13045_2017_502_MOESM3_ESM.docx]

Figure S2.

A.

B.

C.

D.
