# Supplementary material for: Haploidentical allograft is superior to matched sibling donor allograft in eradicating pre-transplantation minimal residual disease of AML patients as determined by multiparameter flow cytometry: a retrospective and prospective analysis
Source: J Hematol Oncol. 2017 Jul 4;10:134. doi: 10.1186/s13045-017-0502-3 (PMC5496245; doi:10.1186/s13045-017-0502-3)
Supplement: Supplementary file 8 — Multivariate analysis of factors associated with outcomes of patients with pre-transplantation MRD-positive who underwent allo-SCT in the prospective study (n = 76). (DOCX 19 kb) [file 13045_2017_502_MOESM8_ESM.docx]

**Table S2**. Multivariate analysis of factors associated with outcomes of patients with pre-transplantation MRD positive who underwent allo-SCT in the prospective study (n=76)

| Covariate | Univariate analysis | | |  | Multivariate analysis | | |
| --- | --- | --- | --- | --- | --- | --- | --- |
|  | HR | 95% CI | *P*-value |  | HR | 95% CI | *P*-value |
| Relapse |  |  |  |  |  |  |  |
| Disease status | 6.808 | 2.348-19.736 | ﹤0.001 |  | 7.304 | 2.389-20.710 | ﹤0.001 |
| Transplant modality | 0.301 | 0.105-0.862 | 0.025 |  | 0.233 | 0.075-0.721 | 0.012 |
| FLT3-ITD (yes vs. no) | 2.661 | 0.832-8.507 | 0.099 |  | 3.578 | 1.034-12.383 | 0.044 |
| Donor-recipient sex matched grafts |  |  |  |  |  |  |  |
| Female-female | 4.899 | 0.896-26.794 | 0.067 |  |  |  |  |
| Female-male | 0.330 | 0.030-3.638 | 0.365 |  |  |  |  |
| Male-male | 2.372 | 0.492-11.427 | 0.282 |  |  |  |  |
| Male-female |  | 1 |  |  |  |  |  |
| Transplant-related mortality |  |  |  |  |  |  |  |
| Age | 7.070 | 0.851-58.751 | 0.070 |  |  |  |  |
| Leukemia-free survival |  |  |  |  |  |  |  |
| Donor-recipient sex matched grafts |  |  |  |  |  |  |  |
| Female-female | 4.965 | 0.908-27.140 | 0.064 |  |  |  |  |
| Female-male | 1.317 | 0.241-7.193 | 0.750 |  |  |  |  |
| Male-male | 3.783 | 0.838-17.080 | 0.084 |  |  |  |  |
| Male-female |  | 1 |  |  |  |  |  |
| Disease status | 4.780 | 2.018-11.318 | ﹤0.001 |  | 5.616 | 2.257-13.971 | ﹤0.001 |
| Transplant modality | 0.325 | 0.138-0.767 | 0.010 |  | 0.221 | 0.085-0.574 | 0.002 |
| Overall survival |  |  |  |  |  |  |  |
| Donor-recipient sex matched grafts |  |  |  |  |  |  |  |
| Female-female | 8.328 | 0.865-80.148 | 0.067 |  |  |  |  |
| Female-male | 2.170 | 0.226-20.866 | 0.502 |  |  |  |  |
| Male-male | 6.749 | 0.854-53.313 | 0.070 |  |  |  |  |
| Male-female |  | 1 |  |  |  |  |  |
| Transplant modality | 0.402 | 0.149-1.081 | 0.071 |  | 0.325 | 0.111-0.952 | 0.040 |
| Time to neutrophil engraftment | 2.654 | 0.855-8.234 | 0.091 |  |  |  |  |
| Disease status | 3.267 | 1.215-8.780 | 0.019 |  | 3.684 | 1.319-10.283 | 0.013 |

**Abbreviations:** HR=hazard ratio; CI=confidence interval; MRD=minimal residual disease; allo-SCT=allogeneic stem cell transplantation

* All variables were first included in the univariate analysis; only variables with *P* < 0.1 were included in the Cox proportional hazards model with time-dependent variables.
