# Supplementary material for: Haploidentical allograft is superior to matched sibling donor allograft in eradicating pre-transplantation minimal residual disease of AML patients as determined by multiparameter flow cytometry: a retrospective and prospective analysis
Source: J Hematol Oncol. 2017 Jul 4;10:134. doi: 10.1186/s13045-017-0502-3 (PMC5496245; doi:10.1186/s13045-017-0502-3)
Supplement: Supplementary file 10 — Multivariate analysis of factors associated with outcomes of patients with pre-transplantation MRD-positive who underwent allo-SCT both in the retrospective study and the prospective study categorization in two groups according to MRD load and transplant modalities (n = 141). (DOCX 21 kb) [file 13045_2017_502_MOESM10_ESM.docx]

**Table S3**. Multivariate analysis of factors associated with outcomes of patients with pre-transplantation MRD positive who underwent allo-SCT both in the retrospective study and the prospective study categorization in two group according to MRD load and transplant modalities (n=141).

| Covariate | Univariate analysis | | |  | Multivariate analysis | | |
| --- | --- | --- | --- | --- | --- | --- | --- |
|  | HR | 95% CI | *P*-value |  | HR | 95% CI | *P*-value |
| Relapse |  |  |  |  |  |  |  |
| Patient subgroup (Group A) |  |  |  |  |  |  |  |
| Transplant modality (Haplo-SCT vs. MSDT) | 0.254 | 0.092-0.702 | 0.008 |  | 0.366 | 0.127-1.056 | 0.063 |
| Patient subgroup (Group B) |  |  |  |  |  |  |  |
| Transplant modality (Haplo-SCT vs. MSDT) | 0.281 | 0.097-0.816 | 0.020 |  | 0.208 | 0.061-0.701 | 0.011 |
| Disease status (CR1 vs. CR﹥1) | 4.781 | 2.301-9.936 | ﹤0.001 |  | 5.142 | 2.341-11.294 | ﹤0.001 |
| Chronic GVHD (yes vs. no) | 0.284 | 0.125-0.646 | 0.003 |  | 0.322 | 0.129-0.803 | 0.015 |
| Platelet engraftment | 0.154 | 0.019-1.220 | 0.076 |  |  |  |  |
| FLT3-ITD (yes vs. no) | 3.937 | 1.656-9.358 | 0.002 |  | 3.466 | 1.260-9.536 | 0.016 |
| Transplant-related mortality |  |  |  |  |  |  |  |
| Recipient age | 5.252 | 1.151-23.976 | 0.032 |  | 4.911 | 1.072-22.499 | 0.040 |
| Neutrophil engraftment | 10.555 | 1.362-81.817 | 0.024 |  |  |  |  |
| Platelet engraftment | 0.010 | 0.001-0.106 | ﹤0.001 |  | 0.011 | 0.001-0.124 | ﹤0.001 |
| Leukemia-free survival |  |  |  |  |  |  |  |
| Patient subgroup (Group A) |  |  |  |  |  |  |  |
| Transplant modality (Haplo-SCT vs. MSDT) | 0.379 | 0.159-0.900 | 0.028 |  | 0.243 | 0.095-0.650 | 0.004 |
| Patient subgroup (Group B) |  |  |  |  |  |  |  |
| Transplant modality (Haplo-SCT vs. MSDT) | 0.253 | 0.104-0.616 | 0.002 |  | 0.438 | 0.168-1.140 | 0.091 |
| Disease status (CR1 vs. CR﹥1) | 3.632 | 1.947-6.775 | ﹤0.001 |  | 3.979 | 1.986-7.972 | ﹤0.001 |
| Neutrophil engraftment | 0.044 | 0.005-0.363 | 0.004 |  |  |  |  |
| Platelet engraftment | 0.059 | 0.016-0.215 | ﹤0.001 |  | 0.127 | 0.030-0.527 | 0.004 |
| Chronic GVHD (yes vs. no) | 0.244 | 0.119-0.501 | ﹤0.001 |  | 0.254 | 0.118-0.547 | ﹤0.001 |
| FLT3-ITD (yes vs. no) | 2.955 | 1.350-6.466 | 0.007 |  | 3.466 | 1.260-9.536 | 0.016 |
| Overall survival |  |  |  |  |  |  |  |
| Patient subgroup (Group A) |  |  |  |  |  |  |  |
| Transplant modality (Haplo-SCT vs. MSDT) | 0.365 | 0.144-0.928 | 0.034 |  | 0.476 | 0.174-1.297 | 0.146 |
| Patient subgroup (Group B) |  |  |  |  |  |  |  |
| Transplant modality (Haplo-SCT vs. MSDT) | 0.348 | 0.132-0.919 | 0.033 |  | 0.213 | 0.073-0.618 | 0.004 |
| Disease status (CR1 vs. CR﹥1) | 3.139 | 1.594-6.182 | 0.001 |  |  |  |  |
| Neutrophil engraftment | 0.007 | 0.001-0.115 | ﹤0.001 |  |  |  |  |
| Platelet engraftment | 0.020 | 0.004-0.092 | ﹤0.001 |  | 0.027 | 0.006-0.133 | ﹤0.001 |
| FLT3-ITD (yes vs. no) | 2.969 | 1.282-6.877 | 0.011 |  | 2.701 | 1.109-7.158 | 0.046 |

**Abbreviations:** MSDT=human leukocyte antigen-matched sibling donor transplantation; HR=hazard ratio; CI=confidence interval; EBMT=European Group for Blood and Marrow Transplantation

Group A = patients with a detectable MRD load less than the quantitative range (＜10^-2^ leukemic cells; n = 86); Group B = patients with MRD load between ≥10^-2^ leukemic cells (n=55).

* All variables were first included in the univariate analysis; only variables with *P* < 0.1 were included in the Cox proportional hazards model with time-dependent variables.
