# Supplementary material for: Haploidentical allograft is superior to matched sibling donor allograft in eradicating pre-transplantation minimal residual disease of AML patients as determined by multiparameter flow cytometry: a retrospective and prospective analysis
Source: J Hematol Oncol. 2017 Jul 4;10:134. doi: 10.1186/s13045-017-0502-3 (PMC5496245; doi:10.1186/s13045-017-0502-3)
Supplement: Supplementary file 12 — Multivariate analysis of factors associated with outcomes of patients with pre-transplantation MRD-positive who underwent allo-SCT both in the retrospective study and the prospective study without receiving donor lymphocyte infusion (n = 105). (DOCX 19 kb) [file 13045_2017_502_MOESM12_ESM.docx]

**Table S4**. Multivariate analysis of factors associated with outcomes of patients with pre-transplantation MRD positive who underwent allo-SCT both in the retrospective study and the prospective study without receiving donor lymphocyte infusion (n=105)

| Covariate | Univariate analysis | | |  | Multivariate analysis | | |
| --- | --- | --- | --- | --- | --- | --- | --- |
|  | HR | 95% CI | *P*-value |  | HR | 95% CI | *P*-value |
| Relapse |  |  |  |  |  |  |  |
| Disease status (CR1 vs. CR﹥1) | 6.714 | 1.931-23.342 | 0.003 |  | 14.090 | 3.260-60.895 | ＜0.001 |
| Transplant modality | 0.160 | 0.045-0.568 | 0.005 |  | 0.100 | 0.024-0.425 | 0.002 |
| Chronic GVHD (yes vs. no) | 0.178 | 0.037-0.849 | 0.030 |  | 0.070 | 0.011-0.435 | 0.004 |
| FLT3-ITD (yes vs. no) | 5.925 | 1.480-23.723 | 0.012 |  |  |  |  |
| Transplant-related mortality |  |  |  |  |  |  |  |
| Recipient age | 7.659 | 0.942-62.266 | 0.057 |  |  |  |  |
| Disease status (CR1 vs. CR﹥1) | 3.543 | 0.846-14.847 | 0.084 |  |  |  |  |
| Neutrophil engraftment | 0.010 | 0.001-0.154 | 0.001 |  |  |  |  |
| Platelet engraftment | 0.008 | 0.001-0.087 | ＜0.001 |  | 0.008 | 0.001-0.087 | ＜0.001 |
| EBMT score |  |  |  |  |  |  |  |
| 0-1 | 0.095 | 0.009-1.048 | 0.055 |  |  |  |  |
| 2 | 0.043 | 0.004-0.475 | 0.010 |  |  |  |  |
| 3 | 0.123 | 0.017-0.876 | 0.036 |  |  |  |  |
| 4 | 0.345 | 0.048-2.458 | 0.001 |  |  |  |  |
| 5 |  | 1 |  |  |  |  |  |
| Leukemia-free survival |  |  |  |  |  |  |  |
| Disease status (CR1 vs. CR﹥1) | 5.288 | 2.080-13.445 | ＜0.001 |  | 8.761 | 2.940-26.107 | ＜0.001 |
| Neutrophil engraftment | 0.039 | 0.004-0.349 | 0.004 |  |  |  |  |
| Platelet engraftment | 0.042 | 0.008-0.217 | ＜0.001 |  | 0.082 | 0.013-0.508 | 0.007 |
| Transplant modality | 0.292 | 0.115-0.742 | 0.010 |  | 0.161 | 0.055-0.470 | 0.001 |
| Chronic GVHD (yes vs. no) | 0.146 | 0.042-0.507 | 0.002 |  | 0.084 | 0.021-0.339 | ＜0.001 |
| Overall survival |  |  |  |  |  |  |  |
| Disease status (CR1 vs. CR﹥1) | 4.248 | 1.615-11.179 | 0.003 |  | 3.648 | 1.355-9.823 | 0.010 |
| Neutrophil engraftment | 0.010 | 0.001-0.154 | 0.001 |  |  |  |  |
| Platelet engraftment | 0.025 | 0.005-0.141 | ＜0.001 |  | 0.017 | 0.003-0.112 | ＜0.001 |
| Transplant modality | 0.352 | 0.134-0.927 | 0.035 |  | 0.323 | 0.115-0.908 | 0.032 |

**Abbreviations:** MSDT=human leukocyte antigen-matched sibling donor transplantation; HR=hazard ratio; CI=confidence interval; EBMT=European Group for Blood and Marrow Transplantation

* All variables were first included in the univariate analysis; only variables with *P* < 0.1 were included in the Cox proportional hazards model with time-dependent variables.
